# Societal values versus individual rights with respect to public health related practices during the COVID-19 pandemic in the U.S.

**DOI:** 10.1371/journal.pone.0358741

**Published:** 2026-09-24

**Authors:** Courtney Bir, Nicole Olynk Widmar, Christopher A. Wolf, Torrie Sheridan, Maria Berikou

**Affiliations:** 1 Department of Agricultural Economics, Oklahoma State University, Stillwater, Oklahoma, United States of America; 2 Department of Agricultural Economics, Purdue University, West Lafayette, Indiana, United States of America; 3 Charles H. Dyson School of Applied Economics and Management, Cornell University, Ithaca, New York, United States of America; 4 Purdue University Brand Studio, Purdue University, West Lafayette, Indiana, United States of America; Touro University California College of Pharmacy, UNITED STATES OF AMERICA

## Abstract

Policies aimed at limiting COVID-19 spread during the pandemic generated dissatisfaction and resistance as they limited privileges or were perceived to infringe on individuals’ rights. Simultaneously, pandemic control and risk mitigation practices are seemingly subject to social resistance, just as other personal risk mitigating practices like seat belt use are. This analysis contributes to understanding U.S. resident perceptions of and compliance with public health pandemic control measures in January 2021. U.S. resident agreement with societal values statements as they pertain to healthcare, societal obligations to protect the elderly and children, and individual rights were studied. For all personal viral spread reducing behaviors studied, likelihood of participation increased with increasing agreement that: healthcare is a human right; the respondent wears a seat belt; we have a societal responsibility to protect the elderly; and having themselves or someone in their household in the higher risk group for COVID-19 complications. Agreement that gun ownership was a right decreased the likelihood of participation in any of the personal behaviors that affect viral spread. This study aims to understand societal perceptions of individual responsibility and rights relative to societal values and responsibilities, which has implications for future public health policy discussions.

## Introduction

On March 11, 2020, the World Health Organization (WHO) characterized the COVID-19 outbreak as a pandemic [1]. In response, most governments adopted unusual measures and placed restrictions on some individual rights and/or behaviors, such as limiting freedom of movement. The general aim was reduction of human-to-human novel coronavirus transmission and to protect other rights, such as the right to health [2,3]. Public health policy actions are frequently needed to fight illness and protect population health as part of the worldwide response to public health emergencies like pandemics [4]. As Van Kolfschooten and de Ruijter (2020) mentioned “*when disease becomes a threat to security, the balance between the need to fight the disease and obligation to protect the rights of individuals often changes*” (p.478) [5].

The COVID-19 toll on the community, economy and health in the U.S. was among the highest in the world [6]. More people were impacted by financial and social pressures, such as unemployment and isolation, in a shorter amount of time than by any previous economic crisis or natural disaster [7]. Disaster-induced stress is well-documented with respect to natural disasters and traumatic events, but pandemic-related stress and then “COVID fatigue” are also concerns for mental and public health [8].

According to Nay (2020), big crises that produce societal shocks might eventually prompt a positive rethinking of the common good and essential rights [9]. Cheng et al. (2022) pointed out that this measure puts emphasis on altruism instead of self-preservation, and serves as a symbol of social solidarity in the international response to the pandemic [10]. For example, wearing a mask can be set side by side with safe driving. If everyone drives cautiously, the benefits of safe driving extend to pedestrians and other road users, and the likelihood of traffic accidents is decreased.

Governments have the obligation to protect women, the elderly, individuals with disabilities, and children from violence and abuse, as well as guarantee the continuation of support services [11]. Governments will violate human rights obligations if they do not take action against communicable diseases, such as enacting disease control measures [12]. Although the virus poses a threat to the rights to life and health, the crisis’ effects on human rights extends far beyond issues of medicine and public health [1]. The COVID-19 global health crisis wasn’t just a health emergency, but also an economic, social and human crisis [13]. As the WHO Director-General stated “*All countries must strike a fine balance between protecting health, minimizing economic and social disruption, and respecting human rights*” [1].

Based on the recommendations of public health officials, governments mostly adhered to similar pandemic measures, such as isolation and quarantine, school closings and restrictions on public gatherings [14]. Measures including face mask requirements, social gathering restrictions and border and school closures were effective at preventing the spread of COVID-19 [7]. Beginning in the middle of March 2020, the United States adopted global practices and quarantine guidance in an effort to “flatten the curve” and limit the spread of infectious disease. Although mask use can reduce the transmission of COVID-19, it was nevertheless controversial in the U.S. [15].

In early 2021 the COVID-19 pandemic raged as fatigue from social distancing and other precautions [16] wreaked havoc during the U.S. winter holiday season. In December 2020 holidays were different, distanced and overshadowed with concerns for gathering safely [17]. Some family and community members clashed on what was necessary to restore normalcy versus protect community health. By New Year’s 2021 hope had arrived in the form of highly effective vaccines that achieved emergency use approval in the U.S. in December 2020 [18,19]. After which, July 4, 2021, emerged as the national goalpost of some form of normalcy [20], even as December posted to-date record deaths from the pandemic [21]. A concerning possible outcome of exhaustion and the stress of constant coping is carelessness in personal protection behaviors.

This analysis contributes to understanding U.S. resident perceptions of and compliance with public health pandemic control measures in January 2021, including mask wearing, intentions towards or taking of vaccination(s), social distancing, and reducing unnecessary interactions and travel. U.S. resident agreement with societal values statements as they pertain to healthcare, societal obligations to protect the elderly and children, and individual rights are studied. It is hypothesized that individual experiences with COVID-19 losses, including the death of loved ones, loss of economic stability, and/or loss of pre-COVID-19 routines (i.e., loss of childcare) may impact willingness to engage in pandemic risk-mitigating behaviors. Specific objectives were to evaluate the impact of COVID-19 on individual lifestyles, the likelihood of participating in behaviors that may impact the spread of COVID-19, and then to evaluate those choices through the lens of societal value statements. By addressing perceptions of risk alongside mitigating practices and self-reported societal values, this work seeks to understand the diversity of societal costs associated with the pandemic-era, including health, social and cultural.

## Methods

### Data collection methods

Qualtrics, an online survey platform [22], was used to create an online survey to evaluate U.S. resident perceptions of COVID-19, pandemic impacts on their lives. The questionnaire included 6 demographic related questions including male or female, age, number of adults and children in the household, annual pre-tax income, education level, and region of residence. Next, respondents were asked to indicate on a scale from 1 (not impacted) to 5 (impacted) how impacted they were by COVID-19 for 8 statements. Statements included: *ability to buy paper products (e.g., toilet paper, paper towels)*; *ability to find meat, milk, and perishable grocery items*; *ability to execute travel plans*; *illness in my family/household from COVID-19*; *death of family member(s) from COVID-19*; *lack of childcare impacted my ability to work*; *I took on education-related responsibilities for my child*; and *I took on care responsibilities for an adult member of my family*. The lifestyle statements were chosen based on news media and popular media discussion surrounding the challenges faced those within the U.S [23,24]. A wide range of challenges were included to reflect challenges faced by different age groups and living arrangements, for example- one may assume those with children felt a large impact due to lack of childcare [25]. However, those without children may have been more greatly impacted by the inability to find paper products [26] or other grocery items [27,28]. Given the uncertainty between media noise and people’s actual experiences the range of impacts were included.

The option “does not apply to me” was included.

Next, respondents were also asked to indicate on a scale of 1 (extremely unlikely) to 5 (extremely likely) how likely they were to participate in personal behaviors that may impact the spread of COVID-19. Personal behaviors included: *wear a mask or face covering in public*; *reduce number of non-essential errands/interactions around town*; *reduce out-of-town travel*; *comply with government orders regarding closures or lock downs*; and *comply with government recommendations regarding social distancing*. These personal behaviors were adapted for the questionnaire from the CDC’s summary of guidance for public health strategies that included recommendations to minimize the spread of COVID-19 [29].

Social value statements were presented to respondents who were asked to indicate their level of agreement from 1 (strongly disagree) to 5 (strongly agree). These statements included: *Gun ownership is a right based on the U.S. Constitution; Healthcare is a human right; I always wear my seat belt when driving; I frequently drink alcohol; I frequently smoke; I believe we have a societal responsibility to protect children; I believe we have a societal responsibility to protect the elderly; Someone in my household or that I frequently spend time with is at higher risk of complications of COVID-19;* and *I am in the higher risk group for complications of COVID-19*. These statements were taken directly from Bir and Widmar (2021) [30].

Finally, respondents were asked to indicate if they had experienced a divorce, new marriage, moving/relocation, death of a household or family member, serious illness of household or family member, start of a new job, loss of job, serious financial distress, pregnancy or purchase of a new home since March of 2020. Pretesting occurred internally with university personnel who were experienced with survey design. The questionnaire and corresponding data set is provided alongside the manuscript.

### Sample characteristics

Survey respondents were part of an opt-in online panel. The survey was only administered online. All survey respondents were required to be 18 years or older to participate. Respondents were provided with information about the project and indicated consent to participate by clicking the arrow at the end of the consent information to enter the survey (see supplemental information for complete consent form text and survey questions). Quotas were set in Qualtrics to target the proportion of respondents to match the U.S. census proportions for sex, age, education, income and U.S. region of residence [31,32].

To evaluate statistical differences between the proportions of respondents in sample demographic categories and the U.S. census, the test of proportions was employed. The test of population proportion, assuming a normal distribution, is given as:


$$\begin{array}{c} z=\frac{\hat{P}-p_{\text{0}}}{\sqrt{\frac{p_{\text{0}}(\text{1}-p_{\text{0}})}{n}}} \end{array}$$ (1)

where *p*_*0*_ is the hypothesized proportion (in this analysis, the census percentage), $\hat{P}$ is the sample proportion, and *n* is the sample size [33]. Equation 1 was used to compare the sample to the U.S. population. A test of the difference of two proportions $\hat{p_{\text{1}}}$ and $\hat{p_{\text{2}}}$, for example, comparing the demographics within a subsample, can be calculated as:


$$\begin{array}{c} z=\frac{\hat{p_{\text{1}}}-\hat{p_{\text{2}}}}{\sqrt{\hat{p_{p}}\left(\text{1}-\hat{p_{p}}\right)\left(\frac{\text{1}}{n_{\text{1}}}+\frac{\text{1}}{n_{\text{2}}}\right)}} \end{array}$$ (2)

given:


$$\begin{array}{c} \hat{P_{p}}=\frac{x_{\text{1}}+x_{\text{2}}}{n_{\text{1}}+n_{\text{2}}} \end{array}$$ (3)

where *x*_*1*_ and *x*_*2*_ are the total number of successes in the two populations [33]. The tests of proportion were conducted using Stata SE16 [34].

Nine hundred and twenty nine respondents completed the survey after quotas and the 18 and over screener. Respondent demographics closely matched the U.S. population (Table 1). The data used in this analysis is provided in supplemental information (as a downloadable Excel sheet with codebook provided as separate file). The survey sample had fewer respondents aged 18–24 (8%) and 25–34 (13%) when compared to the U.S. population, 12% and 18%, respectively. The sample had a larger percentage of respondents aged 65 and older (23%) when compared to the U.S. population (21%). The proportion of respondents in each income bracket were not statistically different than the U.S. population. A lower percentage of respondents did not graduate from high school (2% vs 11%), and a higher percentage of respondents attended college and earned a bachelor’s degree (34%) and attended college and earned a graduate or professional degree (15%) when compared to the U.S. census of 29% and 13%, respectively. The proportion of respondents from each of the four regions did not differ from the U.S. census with the exception of West, which had a statistically lower percentage of respondents at 18% when compared to the U.S. population (24%). The average household in the sample consisted of 2.03 adults, and 25% of respondents had a child under the age of 18 in the home.

**Table 1:** Demographic information, n = 929.

Demographic Variable	Percentage of Respondents	US Census
*Gender*		
Male	46	49
Female	54	51
*Age*		
18-24	8^ψ^	12
25-34	13 ^ψ^	18
35-44	19 ^ψ^	16
45-54	16	16
55-65	20 ^ψ^	17
65 +	23	21
*Income*		
$0-$24,999	19	18
$25,000-$49,999	22	20
$50,000-$74,999	16	17
$75,000-$99,999	13	13
$100,000 and higher	29	31
*Education*		
Did not graduate from high school	2 ^ψ^	11
Graduated from high school, Did not attend college	27	27
Attended college, No degree earned	21	21
Attended college, Associates or bachelor’s degree earned	34 ^ψ^	29
Attended college, Graduate or professional degree earned	15 ^ψ^	13
*Region of residence*		
Northeast	18	17
South	41	38
Midwest	22	21
West	18 ^ψ^	24
*Household make-up*	Average number	
Adults (over 18 years) n = 911^1^	2.04	
Children ages 0–4 n = 751	0.16	
Children ages 5–10 n = 764	0.26	
Children ages 11–15 n = 768	0.24	
Children ages 16–18 n = 743	0.13	

ψ Indicates the percentage of respondents is statistically different than the U.S. census at the 0.05 level.

^1^not all respondents indicated their household makeup, n is as given.

### Sample administration

Data collection occurred from January 13, 2021 through January 23, 2021. Kantar, a company that hosts online panels, was used to obtain survey respondents [35].

### Ethical considerations

Respondents were provided with information about the project and indicated consent to participate by clicking the arrow at the end of the consent information to enter the survey (see supplemental information for consent form text and survey questions used). No identifiable information was collected by researchers and respondents could quit the survey at any time. This survey was approved by Purdue IRB number IRB-2021–36.

### Statistical analysis

For the eight lifestyle statements, and the 5 personal behavior statements the percentage of respondents who selected each impact level option were presented for each statement. Additionally, the mean score for each statement was calculated after excluding those who selected *does not apply to me*. The mean scores of each of the eight statements were statistically compared using a *t*-test. The rank was determined based on the statistical comparison. If a number was statistically higher it was ranked higher, ties occurred when there was no statistical difference. The test for µ_x_ (sample x) =µ_y_ (sample y) for unknown σ_x_ (standard deviation) and σ_y_ and σ_x_ ≠ σ_y_ is [36]:


$$\begin{array}{c} t=\frac{\left(\overset{―}{x}-\overset{―}{y}\right)}{{\left(\frac{S_{x}^{\text{2}}}{n_{x}}+\frac{S_{y}^{\text{2}}}{n_{y}}\right)}^{\frac{\text{1}}{\text{2}}}} \end{array}$$ (4)

where $\overset{―}{x}$ is the mean of sample *x*, $\overset{―}{y}$ is the mean for sample *y*, *s* is the standard deviation, and *n* is the sample size. The result of Equation 4 has a Student’s *t*-distribution with *v* degrees of freedom.

For the societal value statements, mean scores were statistically compared to the results of Bir and Widmar (2021) [30], who asked the same set of society-related statements in June 2020 to a nationally representative sample of U.S. residents.

It was hypothesized that the impact of COVID-19 on the lifestyle factors are correlated, so a seemingly unrelated regression (SUR) was used to estimate the relationship between the impact of COVID-19 on respondent lifestyle statements and respondent demographics [37]. If there is no correlation between the error terms across equations, a seemingly unrelated regression collapses to an ordinary least squares estimator. By using SUR efficiency is improved over OLS while the ease of interpretation and other beneficial properties of OLS remain. The Breusch-Pagan test of independence was used to evaluate the appropriateness of the seemingly unrelated regression [38]. The test indicated that the models were correlated with a p-value of <0.001, indicating that a seemingly unrelated regression was an appropriate estimation method. Models for the eight lifestyle statements were specified as:


$$\begin{array}{c} Lifestylestatementscore=\beta_{\text{1}}+\beta_{\text{2}}Male+\beta_{\text{3}}Age+\beta_{\text{4}}Income+\beta_{\text{5}}Northeast+\beta_{\text{6}}South\\ +\beta_{\text{7}}Midwest+\beta_{\text{8}}KidYoung+\beta_{\text{9}}KidSchool+\beta_{\text{1}\text{0}}Rurality+\varepsilon \end{array}$$ (5)

where β_1_ is the intercept, *Male* is the male sex as opposed to female, *Age* is a continuous variable for age of the respondent, and *Income* is a continuous variable for the respondent’s reported income. *Northeast*, *South*, and *Midwest* indicate the respondent’s region of residence and is in comparison to the dropped region of residence *West*. The region West was dropped to avoid multicollinearity [37]. Regions of residence match that of the US census. The region Northeast includes the states CT, ME, MA, NH, NJ, PA, RI, VT; South includes the state AL, AR, DE, DC, FL, GA, KY, LA, MD, MS, NC, OK, SC, TN, VA, WV; Midwest includes IL, IN, IA, KS, MI, MN, MO, NE, ND, OH, SD, WI; West includes AK, AZ, CA, CO, HI, ID, MT, NV, NM, OR, UT, WA, WY.

Individual experiences during the pandemic varied, as well as regional laws/experiences. Although most residents of the US experienced closures, masking etc at some point, those in different regions such as the South and Midwest experienced shorter and less restrictive closures. Therefore, we predicted regional differences within this analysis. *KidYoung* indicates the respondent had a child aged 4 or younger in the household, and *KidSchool* indicates the respondent had a child aged 5 or older in the household. These two variables were included only in the estimation for the statements, *lack of childcare impacted my ability to work* and *I took on education-related responsibilities for my child.* The level of rurality (*Rurality*) was determined using the respondent county of residence and the USDA rural-urban continuum codes. The USDA has nine rural-urban continuum code categories ranging from 1 (counties in metro areas of 1 million population or more) to 9 (completely rural or less than 2,500 urban population, not adjacent to a metro area) [39]. ϵ is the error term.

A second seemingly unrelated regression was employed to evaluate the relationship between the personal behaviors that may impact the spread of COVID-19, demographics, social beliefs and the impact of COVID-19 on lifestyle. Again, the Breusch-Pagan test of independence was employed, and the models were correlated with a p-value of <0.001 [38]. Models were specified as:


$$\begin{array}{c} Personalbehaviorscore=\beta_{\text{1}}+\beta_{\text{2}}Male+\beta_{\text{3}}Age+\beta_{\text{4}}Income+\beta_{\text{5}}Rurality+\beta_{\text{6}}BuyPaper\\ +\beta_{\text{7}}FindGrocery+\beta_{\text{8}}Travel+\beta_{\text{9}}Illness+\beta_{\text{1}\text{0}}Death+\beta_{\text{1}\text{1}}SS\text{1}+\beta_{\text{1}\text{2}}SS\text{2}\\ +\beta_{\text{1}\text{3}}SS\text{3}+\beta_{\text{1}\text{4}}SS\text{4}+\beta_{\text{1}\text{5}}SS\text{5}+\beta_{\text{1}\text{6}}SS\text{6}+\beta_{\text{1}\text{7}}SS\text{7}+\beta_{\text{1}\text{8}}SS\text{8}+\beta_{\text{1}\text{9}}SS\text{9}+\mu \end{array}$$ (6)

where β_1_ is the intercept, *Male* indicates sex, *Age* is a continuous variable for age of the respondent, and *Income* is a continuous variable for the respondent’s reported income. *Rurality* was the rurality score. The COVID-19 impact level questions from 1 (not impacted) to 5 (impacted) are represented by *BuyPaper* (impact level of COVID-19 on ability to buy paper products), *FindGrocery* (impact level of COVID-19 on ability to find meat, milk, and perishable grocery items), *Travel* (impact level of COVID-19 on ability to execute travel plans), *Illness* (impact level of COVID-19 illness in family/household), and *Death* (impact level of COVID-19 death of family member(s)). *BuyPaper* and *FindGrocery* were included only in the models for *Reduce non-essential errands/interactions around town* and *Comply with governmental orders regarding closures or lock downs*. It was hypothesized that those who were reducing non-essential errands/interactions and complying with lock down orders would also experience greater difficulty in securing paper products and finding groceries. *Travel* was included only in the model for *Reduce out-of-town travel*. It was hypothesized that having difficulty executing travel plans would result in a reduction of out-of-town travel. All nine social value statements starting with *SS1* (*Gun ownership is a right* *based on the U.S. constitution.)* to *SS9* (*I am in the higher risk group for complications of COVID-19.)* were included for all models of personal behavior.

## Results

The following results evaluate COVID-19’s impact on individual’s lifestyles, participation in behaviors to mitigate the spread of COVID-19, and then the evaluation of these components through the lens of societal values. When evaluating the impact of COVID-19 on the respondent lifestyle, *ability to execute travel plans* had the highest mean impact (3.29) (Table 2). There was a three-way tie for the second most impacted between *ability to buy paper products (e.g., toilet paper, paper towels)* (2.51), *taking on education-related responsibilities for my child* (2.61), and *taking on care responsibilities for an adult member of my family* (2.45). *Lack of childcare impacting ability to work* was ranked third out of four in order of most to least impacted with a mean of (2.26). *Ability to find meat, milk, and perishable grocery items* (2.51), *illness in my family/household from COVID-19* (2.21), and *death of family member(s) from COVID-19* (2.14) all ranked last in terms of impact.

**Table 2:** Impact of COVID-19 on lifestyle, n = 929.

	Percentage of Respondents							
	1 (Not Impacted)	2	3	4	5 (Impacted)	Does Not Apply to Me	Mean^1^	Rank
Ability to buy paper products (e.g., toilet paper, paper towels)	37	12	15	10	16	11	2.51a^2^ (1.540) n = 829	2
Ability to find meat, milk, and perishable grocery items	44	10	12	10	10	14	2.18b (1.444) n = 798	4
Ability to execute travel plans	18	5	6	8	26	36	3.29d (1.707) n = 591	1
Illness in my family/household from COVID-19	32	5	6	5	9	43	2.21b (1.553) n = 527	4
Death of family member(s) from COVID-19	30	3	3	4	8	52	2.14b (1.604) n = 448	4
Lack of childcare impacted my ability to work	22	2	5	4	6	61	2.26bc (1.563) n = 366	3/4
I took on education-related responsibilities for my child	20	2	6	6	9	57	2.61a (1.648) n = 404	2
I took on care responsibilities for an adult member of my family	24	3	5	6	9	52	2.45ac (1.638) n = 442	2/3

^1^Mean does not include those who selected “Does not apply to me”, n for individual questions given.

^2^Matching letters indicate the means are not statistically different. For example, the mean for ability to buy paper products is not statistically different than the mean for illness in my family/household from COVID-19. Non-matching numbers indicate the means are statistically different. For example, the mean for ability to buy paper products is statistically different than the ability to find meat, milk and perishable grocery items.

In the seemingly unrelated regression of impact of COVID-19 on lifestyle factors, all models were statistically significant as indicated by a p value less than 0.000 for all models and correlated as indicated through the Breusch-Pagan test of independence (Table 3). In the model of ability to buy paper products, increasing age decreased the impact (−0.265), and reporting northern residence (−0.420), southern residence (−0.361) and midwestern residence (−0.425) all decreased impact when compared to the West. The impact on ability to execute travel plans increased for males (0.254) when compared to females, decreased with age (−0.242) and increased with income (0.305). The impact of the death of family member(s) from COVID-19 decreased with age (−0.305) and increased with income (0.111). Being male increased the self-reported impact felt from lack of childcare (0.368). The impact from lack of childcare decreased with age (−0.275), increased with income (0.092). However, having both young children (0.455) and school-aged kids (0.534) increased the impact. Similarly, being male increased the self-reported impact felt from taking on education-related responsibilities for a child (0.236). Impact decreased with age (−0.257) and increased with income (0.083). As rurality increased, the impact of taking on education-related responsibilities decreased (−0.043). Finally, the impact felt from taking on care responsibilities for an adult member of their family increased if the respondent was male (0.298), decreased with age (−0.378), and increased with income (0.089).

**Table 3:** Seemingly unrelated regression results. Impact of COVID-19 on lifestyle factors and demographics. Level of impact from 1 (un-impacted to 5 (impacted) N = 929.

	Independent Variables Coefficient (Standard Error)									
				Region of residence^1^						
Dependent Variables	Male	Age	Income	Northeast	South	Midwest	Young kids	School kids	Rurality	Constant
Ability to buy paper products (e.g., toilet paper, paper towels)	−0.087 (0.107)	−0.265^***^ (0.034)	0.006 (0.035)	−0.420^**^ (0.173)	−0.361^**^ (0.147)	−0.425^**^ (0.167)			−0.028 (0.023)	3.970^***^ (0.223)
Ability to find meat, milk, and perishable grocery items	0.066 (0.098)	−0.296^***^ (0.031)	0.020 (0.032)	−0.396^**^ (0.159)	−0.192 (0.135)	−0.322^**^ (0.153)			−0.010 (0.021)	3.497^***^ (0.205)
Ability to execute travel plans	0.254* (0.136)	−0.242^***^ (0.043)	0.305^***^ (0.045)	−0.165 (0.220)	−0.047 (0.187)	−0.208 (0.212)			−0.035 (0.029)	2.420^***^ (0.284)
Illness in my family/household from COVID-19	0.201* (0.103)	−0.308^***^ (0.033)	0.085^**^ (0.034)	−0.120 (0.167)	0.109 (0.142)	0.039 (0.161)			0.001 (0.022)	2.382^***^ (0.215)
Death of family member(s) from COVID-19	0.162 (0.099)	−0.305^***^ (0.031)	0.111^***^ (0.032)	−0.238 (0.160)	0.067 (0.136)	−0.097 (0.154)			−0.012 (0.021)	2.193^***^ (0.206)
Lack of childcare impacted my ability to work	0.368^***^ (0.086)	−0.275^***^ (0.029)	0.092^***^ (0.028)	−0.315^**^ (0.139)	−0.011 (0.118)	−0.276^**^ (0.133)	0.455^***^ (0.107)	0.534^***^ (0.074)	−0.020 (0.018)	1.774^***^ (0.189)
I took on education-related responsibilities for my child	0.236^**^ (0.092)	−0.257^***^ (0.032)	0.083^**^ (0.031)	−0.392^**^ (0.149)	−0.134 (0.127)	−0.222 (0.144)	0.585^***^ (0.131)	1.197^**^ (0.090)	−0.043^**^ (0.020)	1.922^***^ (0.206)
I took on care responsibilities for an adult member of my family	0.298^***^ (0.104)	−0.378^***^ (0.033)	0.089^**^ (0.034)	−0.077 (0.169)	0.180 (0.144)	−0.109 (0.163)			−0.002 (0.023)	2.588^***^ (0.218)

^1^Coefficients for region of residence are in relation to the dropped region of residence West to prevent multicollinearity.

^2^Young kids indicates the respondent has a child aged 4 or under. School kids indicates the respondent has a child aged 5 or older.

* indicates statistical significance at the 0.10 level, ** at the 0.05 level, *** at the < 0.001 level.

R squared for models from top to bottom 0.0829, 0.1062, 0.0755, 0.0962, 0.1084, 0.2547, 0.3417, 0.1392. All models are statistically significant at the 0.001 level.

Respondents were asked to report from a scale of 1 (extremely unlikely) to 5 (extremely likely) how likely they were to adopt behaviors related to stopping the spread of COVID-19 (Table 4). Wearing a mask or face covering in public had the highest mean score (4.38), indicating it was most likely to be adopted. The next most likely adopted behavior was compliance with government recommendations regarding social distancing (4.19), followed by compliance with governmental orders regarding closures or lock downs (4.05). People were less likely to reduce out-of-town travel (3.86) and reduce the number of non-essential errands/interactions around town (3.70).

**Table 4:** Likelihood of participating in personal behaviors that may impact the spread of COVID-19.

	Likelihood of Participating, Percentage of Respondents					
Personal Behaviors	1 (Extremely Unlikely)	2	3	4	5 (Extremely Likely)	Mean (Standard Deviation)
Wear a mask or face covering in public n = 928	5	3	10	13	69	4.38a^1^ (1.095)
Reduce number of non-essential errands/interactions around town n = 928	11	7	21	23	38	3.70b (1.338)
Reduce out-of-town travel n = 928	10	6	19	16	48	3.86c (1.350)
Comply with governmental orders regarding closures or lock downs n = 927	6	6	17	17	53	4.05d (1.229)
Comply with government recommendations regarding social distancing n = 927	6	4	13	19	58	4.19e (1.165)

^1^Matching letters indicate the means are not statistically different. Differing letters indicate the means are statistically different. All means are statistically different for the personal behaviors.

Level of agreement from 1 (strongly disagree) to 5 (strongly agree) was collected for societal value statements to gain a broader understanding of respondents’ beliefs and values (Table 5). Respondents reported strong agreement that healthcare is a human right (4.10), that we have a societal responsibility to protect children (4.36), and that we have a societal responsibility to protect the elderly (4.27).

**Table 5:** Level of agreement with societal value statements in January 2021, n = 929.

	1 (Strongly Disagree)	2	3	4	5 (Strongly Agree)	Mean (Standard Deviation)	Level of agreement in June 2020 national sample from Bir and Widmar (2021) Mean (SD)
Gun ownership is a right based on the U.S. Constitution	9	8	23	13	47	3.83 (1.326)	3.78 (1.30)
Healthcare is a human right	5	5	19	20	51	4.07 (1.168)	4.01 (1.17)
I always wear my seat belt when driving	3	2	10	10	76	4.53 (0.966)	4.49 (1.01)
I frequently drink alcohol	42	16	19	12	11	2.35^ψ^ (1.410)	2.20 ^ψ^ (1.35)
I frequently smoke	70	3	8	8	12	1.88 (1.457)	1.95 (1.49)
I believe we have a societal responsibility to protect children	4	2	13	18	64	4.36 (1.022)	4.37 (1.00)
I believe we have a societal responsibility to protect the elderly	3	4	15	21	58	4.27 (1.026)	4.25 (1.03)
Someone in my household or that I frequently spend time with is at higher risk of complications of COVID-19	31	11	18	16	25	2.93 (1.578)	2.92 (1.55)
I am in the higher risk group for complications of COVID-19	31	14	19	18	18	2.80 (1.500)	2.86 (1.52)

ψ Indicates mean response is statistically different than Bir and Widmar 2020 at the 0.05 level.

Demographics, level of impact of COVID-19 on daily activities, and responses to the societal value statements were incorporated into a seemingly unrelated regression of likelihood of participating in personal behaviors that may impact the spread of COVID-19 (Table 6). For all behaviors, the likelihood of participation increased with increasing agreement that: healthcare is a human right; the respondent wears a seat belt when driving; we have a societal responsibility to protect the elderly; and having someone in the household they spend time with or the respondent themself is in the higher risk group for complications of COVID-19. Conversely, increasing agreement that gun ownership is a right decreased the likelihood in participating in any of the personal behaviors that may affect the spread of COVID-19. As age increased, the probability of wearing a mask or face covering in public (0.058), complying with government orders regarding closures or lock downs (0.094), and complying with government recommendations for social distancing increased (0.095).

**Table 6:** Seemingly unrelated regression of COVID-19 related precautions and demographics, n = 922.

	Independent Variables Coefficient (Standard Error)				
Dependent Variables	Wear a mask or face covering in public	Reduce number of non-essential errands/ interactions around town	Reduce out-of-town travel	Comply with governmental orders regarding closures or lock downs	Comply with government recommendations regarding social distancing
Male	0.009 (0.067)	−0.121 (0.083)	−0.105 (0.084)	−0.123^*^ (0.071)	−0.071 (0.070)
Age	0.058^**^ (0.024)	0.043 (0.031)	0.032 (0.031)	0.094^***^ (0.026)	0.095^***^ (0.026)
Income	0.036 (0.022)	0.038 (0.028)	0.037 (0.028)	0.044^*^ (0.024)	0.038^*^ (0.023)
Rurality	0.004 (0.014)	−0.001 (0.017)	−0.003 (0.018)	0.023 (0.015)	0.007 (0.015)
Impact level of COVID on ability to buy paper products		0.074^**^ (0.025)		0.017 (0.018)	
Impact level of COVID on ability to find meat, milk, and perishable grocery items		0.009 (0.028)		0.005 (0.021)	
Impact level of COVID on ability to execute travel plans			0.035^**^ (0.016)		
Impact level of COVID-19 illness in family/household	0.023 (0.026)	0.031 (0.033)	0.023 (0.033)	0.067^**^ (0.028)	0.066^**^ (0.027)
Impact level of COVID-19 death of family member(s)	−0.059^**^ (0.027)	0.017 (0.034)	−0.060^*^ (0.034)	−0.049^*^ (0.029)	−0.076^**^ (0.029)
Gun ownership is a right based on the U.S. Constitution	−0.094^***^ (0.025)	−0.139^***^ (0.031)	−0.131^***^ (0.031)	−0.144^***^ (0.027)	−0.082^**^ (0.026)
Healthcare is a human right	0.217^***^ (0.032)	0.259^***^ (0.040)	0.298^***^ (0.041)	0.297^***^ (0.034)	0.248^***^ (0.034)
I always wear my seat belt when driving	0.166^***^ (0.039)	0.147^**^ (0.048)	0.116^**^ (0.048)	0.174^***^ (0.041)	0.178^***^ (0.040)
Frequently drinks alcohol	−0.014 (0.025)	−0.027 (0.031)	−0.011 (0.032)	0.014 (0.027)	−0.005 (0.026)
Frequently smokes	−0.003 (0.026)	0.004 (0.032)	0.030 (0.032)	−0.002 (0.027)	−0.013 (0.027)
Believes we have a societal responsibility to protect children	0.078^*^ (0.044)	0.030 (0.055)	−0.025 (0.056)	0.094^*^ (0.047)	0.045 (0.046)
Believes we have a societal responsibility to protect the elderly	0.107^**^ (0.046)	0.120^**^ (0.057)	0.168^**^ (0.058)	0.133^**^ (0.049)	0.141^**^ (0.048)
Someone in my household or that I frequently spend time with is at higher risk of complications of COVID-19	0.046^**^ (0.023)	0.061^**^ (0.028)	0.067^**^ (0.028)	0.053^*^ (0.024)	0.044^*^ (0.024)
I am in the higher risk group for complications of COVID-19	0.045^*^ (0.025)	0.077^**^ (0.031)	0.095^**^ (0.031)	0.079^**^ (0.027)	0.058^**^ (0.026)
Constant	1.707^***^ (0.238)	1.017^***^ (0.296)	1.272^***^ (0.298)	0.524^**^ (0.254)	1.057^***^ (0.248)

* indicates statistical significance at the 0.10 level, ** at the 0.05 level, *** at the < 0.001 level.

R squared for models from left to right 0.2284, 0.2052, 0.1994, 0.3058, 0.2560. All models are statistically significant at <0.001.

For the reduced number of non-essential errands/interactions and complying with government orders regarding closures or lock down behaviors, the impact level of ability to buy paper products and the impact level of ability to find meat, milk, and perishable grocery items were included. Interestingly, as impact level on ability to buy paper products increased, the likelihood of reducing the number of non-essential errands increased (0.074). Ability to find other grocery items was not statistically significant in either model. The impact of COVID-19 on ability to execute travel plans was included in the model for reduce out of town travel. As impact on travel plans increased, likelihood to reduce out-of-town travel increased (0.035). As the impact level of COVID-19 illness in family/household increased, the likelihood of complying with government recommendations regarding closures or lock downs (0.067) and social distancing increased (0.066). As the impact level of a death of a family member due to COVID-19 increased, the probability of participating in any behavior with the exception of reducing the number of errands decreased. Believing that one has a societal responsibility to protect children increases the probability of complying with governmental orders regarding closures or lockdowns (0.094).

## Discussion

Policies aimed at limiting COVID-19 spread during the pandemic generated dissatisfaction and resistance. Some mitigation techniques interrupt daily life, limit rights and privileges, and have provoked social unrest [40]. For example, in the United States, armed protesters assembled at the Michigan State Capitol to express their displeasure with the mitigation orders [41]. Across Europe, thousands of people demonstrated against new restrictions implemented in response to an increase in illnesses [42]. In the United Kingdom, there were significant protests against the national lockdown with many demonstrators expressing their anger with the COVID-19 limitations [43]. Erev et al. (2020) reviewed two suboptimal reactions to rare but extreme risks, mainly overreaction and underreaction [44]. Overreaction may lead to grocery stockpiling, which fuels anxiety about availability of necessities, whereas underreaction may facilitate disease spread and threaten the stability of healthcare systems [44].

While a great deal of media attention was paid to food supply chains during the pandemic, especially milk supplies [45] and meat markets [46–50] the representative sample of respondents in this analysis were less concerned the ability to find meat, milk, and perishable groceries. While being older was associated with less self-reported impact on abilities to buy paper products and find meat, milk, and perishable grocery items, it is recognized that previous studies have highlighted additional factors such as depression, hope/hopelessness about the future, and fear for future availability as also driving stockpiling behaviors [51]. They also highlight the propensity to stockpile food is related to not only age, but also race, marital and employment statuses, and whether there are children in the household [51]. Given data collection took place in January 2021 when many supply chains had recovered substantially or demonstrated resiliency [52], respondents may have simply not considered themselves to be greatly impacted at that point. In addition to supply chain recovery, consumer adaptation had taken place in the form of accelerated adoption of online grocery shopping in the pandemic period [53]. Overall relative rankings of impacts were consistent with those reported for June 2020 [30].

Older respondents were less impacted by the change in ability to execute travel plans, which may simply reflect fewer travel plans in place between personal and business travel for those age groups compared to younger respondents. Higher income was associated with higher self-reported impacts from COVID-19, which is not reflective of the disproportionate measured impacts felt by the lowest income households [54] and workers with public-facing retail, food service, and related jobs. Yet, self-reported impacts reflect the perception of higher impact, which could reflect the starting point or the perceived magnitude of impact by individuals generally insulated. Those with higher incomes reported more impact from travel disruptions, which is sensical given the higher income levels among frequent travelers. The impacts of a death in the family from COVID-19 decreased with age but increased with household income. Income, again, may reflect self-perceived relative impacts, whereas death may simply reflect who was dying at that stage of the pandemic (January 2021; older residents [55]). Similarly, households with higher incomes reported more impact of lack of childcare, which may simply reflect their use of childcare.

Self-rated impacts of COVID-19 illness in the household or family and death of family member(s) from COVID-19 both were tied for least impactful amongst factors studied. While it is possible that the lack of impact reported from illness and death reflects desensitization [56], it could also reflecting the unequal impacts of the pandemic on U.S. residents in which some families were devastated while others did not experience first-hand impacts of that magnitude. Furthermore, the unfolding of the pandemic, particularly in the early days, differed substantially between states [57], so individual experiences may have varied for a variety of personal or family reasons, or by local geography. The impact of illness of a family member with COVID-19 decreased with age, perhaps reflecting smaller households among older respondents without children in the home. Age has been found to be associated with greater emotional well-being, even while facing prolonged stresses during the COVID-19 pandemic to which older people were more vulnerable, leading a previous study to the conclusion that “older people display notable emotional resilience” [58].

As schools and daycares closed across the United States to encourage distancing, parents of children were increasingly expected to work full-time jobs while both educating and providing care for their children [59]. Women spend more time caring for children and taking care of the home, so this led to increased conflict between the numerous roles expected of women in the workforce [59]. The pandemic strained many families, including challenging people’s abilities to afford basic necessities; lower-income households were especially hard hit [60]. Additionally, lower-income families often face greater difficulty finding childcare than higher-income families, and may face greater challenges with taking time off of paid work to provide child caregiving. Food insecurity, particularly for households with children, worsened during pandemic-era disruptions [61], further challenging households managing pandemic risks alongside childcare and employment or economic pressures. Impacts felt for lack of childcare were higher for those with higher income, likely reflecting the use of childcare by higher income households pre-pandemic [62]. Rurality has been discussed with relation to access to healthcare and health facilities throughout the pandemic. Interestingly, the impact of taking on education-related responsibilities was lower for those in more rural areas, likely reflecting the fact that schools in rural areas faced pressures to remain open [63]. Rural areas likely faced additional challenges with conducting virtual lessons or online schooling, specifically internet reliability and access [64]. Lessened technological capabilities to conduct remote learning may have heightened the difficulty of alternative modes of education in rural areas.

Respondents self-reported drinking more alcohol in January 2021 than was measured in 2020 [30] supporting the growing body of literature reporting concern and urging public health attention on alcohol consumption changes during the COVID-19 era. Schmidt et al. [65] studied early impacts of COVID-19 and found certain segments of the population increased alcohol use early in the pandemic and urged increased research to inform evidence-based rapid response, from a treatment system perspective. Barbosa, Cowell and Dowd [66] compared May 2020 to February 2020 and found people were consuming more alcoholic drinks per day. Further, a greater proportion of people exceeded recommended drinking limits, and more binge drinking was reported for all sociodemographic subgroups they studied, with larger differences in women than men and for Black, non-Hispanic people than White, non-Hispanic people. Increased alcohol consumption fueled health concerns. However, there are also societal costs associated with excessive drinking, including those arising from lower or lost productivity, health care more broadly, and the need for corrective actions [67].

A great deal of attention was placed on mask wearing in the U.S. during the pandemic, fueled by the CDC’s change in directive and polarizing messaging from various sectors of the U.S. and state governments [30]. Although many nations have laws mandating people to wear masks in public areas, Western societies have a considerably higher level of opposition to such laws than Asian societies [68]. The COVID-19 pandemic brought forward the national conflict between individual rights and the good of the general public. Bello and Amzat [69] summarized that COVID-19 disrupted the social order, caused human, economic, and social crises, and ultimately created social instability. Furthermore, they characterized the struggle for power by individuals while facing “devastating” lockdowns [69]. Toebes [70] investigated the tension between individual rights, including physical integrity, family life and public health policies. According to United Nations [11] “*Human rights are key in shaping the pandemic response, both for the public health emergency and the broader impact on people’s lives and livelihoods”* (p.2). Societies with higher Economist Intelligence Unit’s (EIU) democracy index scores appear to be hesitant to give up too much of their personal sovereignty in order to manage a health crisis, which creates an ethical dilemma in which individual freedom replaces the social obligation to reduce the impact of a communicable disease [71].

Acknowledgement of the emergence of social resistance in response to COVID-19 control measures [69] aids in understanding of decision making. For all personal behaviors studied in this analysis, the likelihood of participation increased with increasing agreement that: healthcare is a human right; the respondent wears a seat belt; we have a societal responsibility to protect the elderly; and having someone in the household they spend time with or the respondent themself is in the higher risk group for complications of COVID-19. The mean level of agreement for each societal value statement was statistically compared to a previous data collection by Bir and Widmar [30]. Interestingly, the only mean score statistically different was for the statement, “*I frequently drink alcohol,*” which had a stronger level of agreement in this sample (2.35) when compared to Bir and Widmar [30] (2.20). Agreement that gun ownership was a right decreased the likelihood of participation in any of the personal behaviors that affect viral spread. As individuals faced more difficulty in finding household paper products (i.e., toilet paper), their likelihood of reducing the number of errands and compliance with government orders increased, perhaps because those individuals were seeing firsthand the supply chain functionality challenges of the pandemic, thus internalizing more the weight or personal responsibility of needing to reduce spread. Similarly, direct impact of COVID-19 illness within a household increased likelihood of complying with government recommendations and social distancing protocols.

As defined by the American Psychological Association [72], social resistance is “group opposition to the political, economic, or social actions and policies of a government or society” or “subgroup opposition to the values and strictures of a dominant culture”. Sheveland et al. [73] found that impulsivity and social resistance orientation were negatively associated with seat belt use. Seat belt use offers an interesting comparison practice to many of the pandemic-era self-protection or risk-mitigating behaviors in the sense that participating in seat belt wearing is known to reduce one’s risk or fatality [74], yet, a proportion of the population does not consistently wear (or even consistently does not wear) their seat belt [73]. Social resistance orientation in this context of seat belt use is associated with behaviors representing resistance to oppression and an individual’s perception that they are part of a disadvantaged group [73]. COVID-19 mitigation practices, and facial mask wearing, raise a novel societal discourse in the United States in some ways, and yet, a familiar one in the sense of social resistance framing. Wearing masks was framed as a social contract in which people who complied perceived one another more positively while noncompliance was punished socially [75]. Yet Bir and Widmar [30] found that, while there was potential evidence of altruism in mask wearing, perceiving social pressure to wear masks actually negatively impacted the probability that individuals wore them even amongst those who felt masks had a role in society. Thus, Bir and Widmar [30] suggested that social shaming (akin to social punishment referenced in other works) may not increase masking compliance.

The emotional and mental health toll in addition to the devastating loss of human life — which included the impacts of young children losing parents and the elderly becoming ill and unable to take visitors — has taken a toll [76,77]. Interpersonal support systems became fraught as individual’s experienced overwhelm, and in some cases struggled to maintain connectedness and relationships. Zerbe [78] explained that coining the term pandemic fatigue in her practice was done in hope of recognizing and normalizing bodily and psychological symptoms of those who did not have Covid-19 nor serve as first responders. Zerbe [78] explains the need to pause, take stock, and gain the interpersonal nourishment necessary to persist through pandemic fatigue; this further highlights the value of interpersonal relationships and connectedness.

Given the sample of respondents was from the U.S., our findings should be interpreted within the context of the U.S. culture, society, and institutional structure. One of the larger limitations of this study is the self-reported nature. When facing self-reported surveys, respondents may succumb to social desirability bias and other issues such as inattention. Careful wording of questions and survey length can help mitigate some of this risk. Although there may be some bias from the use of online surveys, given the prevalence of internet access in the US that bias has been somewhat minimized. Additional precautions including quotas to improve representativeness were employed, but there were some demographic categories that were statistically different from the US census. Beliefs and experiences during the COVID pandemic were rapidly evolving and this work represents a particular snapshot in time.

## Conclusions

Given the economic, social, cultural and overall health (both physical and mental) implications of keeping societies ‘closed’ to some degree to protect public health, increased attention on how individual perceptions and viewpoints may impact behaviors with externalities for others within a society is needed. Public acceptance of practices or behaviors intended to reduce disease transmission and protect public health may depend not only on their expected health benefits and the degree to which those are clearly communicated, but also on how individuals perceive their effects on autonomy and individual rights. Facial mask wearing and other viral transmission mitigation personal practices are seemingly subject to social resistance, just as other practices like seat belt use are.

Responses from a survey by a sample of 929 U.S. residents, of which 25% had a child under the age of 18 in the home, indicated that for all personal viral spread reducing behaviors studied, the likelihood of participation increased with increasing agreement that: healthcare is a human right; the respondent wears a seat belt; we have a societal responsibility to protect the elderly; and having themselves or someone in their household in the higher risk group for COVID-19 complications. The viral control practice studied that respondents were most likely to adopt was wearing a mask or face covering in public, followed by compliance with government social distancing recommendations, and then compliance with government orders regarding closures or lock downs. Agreement that gun ownership was a right decreased the likelihood of participation in any of the personal behaviors that affect viral spread. As individuals faced more difficulty in finding household paper products (i.e., toilet paper), their likelihood of reducing the number of errands and compliance with government orders increased, perhaps because those individuals were seeing firsthand the supply chain functionality challenges of the pandemic and thus internalizing more the weight or personal responsibility of needing to reduce spread. Similarly, direct impact of COVID-19 illness within a household increased likelihood of complying with government recommendations and social distancing protocols.

Appreciation of the complexities of individual decision making, including decision making to undertake practices widely known and accepted to benefit that individual by reducing their own risk, must incorporate the consequences to the individual and society at large. While restrictive COVID-19 era practices have now ended, the role of social resistance in compliance with risk-mitigating practices and the willingness to participate in activities that may limit individual freedoms to improve societal outcomes are societal questions that must be grappled with and apply to a variety of societal situations likely faced in coming years.

## Supporting information

S1 File Survey questions with consent text and coding of responses. (DOCX)

S2 File Data for analysis using coding of responses provided in survey document. (XLSX)
