# Supplementary material for: Societal values versus individual rights with respect to public health related practices during the COVID-19 pandemic in the U.S
Source: PLoS One. 2026 Sep 24;21(9):e0358741. doi: 10.1371/journal.pone.0358741 (PMC13606988; doi:10.1371/journal.pone.0358741)
Supplement: Supplementary file 1 [file pone.0358741.s001.docx]

Q1.1 **RESEARCH PARTICIPANT CONSENT FORM** **Holiday Food Shopping** **Dr. Nicole Widmar, Dr. Courtney Bir & Mario Ortez** **Agricultural Economics** **Purdue University** IRB Number:IRB-2021-36

**Key Information**  Please take the time to review this information carefully. This is a research study. Your participation in this study is voluntary which means that you may choose not to participate at any time without penalty or loss of benefits to which you are otherwise entitled. You may ask questions to the researchers about the study whenever you would like. If you decide to take part in the study, be sure you understand what you will do and any possible risks or benefits.

**Thank you for participating in this study. The following contains information about the study and your rights as a research participant.**

This is a web-based survey research study designed to understand 2020 holidays meat buying behavior. Proceeding with the web-based survey will imply your consent to participate in this study. There are about 50 questions asking basic demographic questions, meat buying during the 2020 holiday season and overall 2020 holiday behavior. The survey will take approximately 20 minutes to complete.

**What is the purpose of this study?** The purpose of this study is to understand consumer meat buying behavior during the 2020 holiday season as well as overall holiday celebrations. We would like to enroll about 1,000 respondents in this study.

**What will I do if I choose to be in this study?**  You will be asked questions about your demographics,  individual and/or household meat buying and celebrations during the 2020 holiday season.

**How long will I be in the study?**  The approximate total time commitment for this study about 20 minutes. There will be approximately 50 questions.

**What are the possible risks or discomforts?**  The risks associated with this study are minimal. The risks are not greater than those ordinarily encountered in daily life. Moreover, you may stop the survey at any time. By participating in the study, you are at no greater cost than you would encounter in daily life. Breach of confidentiality is always a risk with data, but we will take precautions to minimize this risk as described in the confidentiality section.

**Are there any potential benefits?**     This research will assist researchers to better understand meat buying during the December holiday season. The findings of this study may lead stakeholders to improve the holiday meat offerings through product offerings. Additionally, it may shed light on potential holiday buying changes from previous years.

**Are there costs to me for participation?** There are no anticipated costs to participate in this research.

**Will information about me and my participation be kept confidential?**   At no point will a data file be constructed in which your name is linked with your responses. The data will be stored by the principal investigators with no intention to destroy the data. The data will only be released in summaries in which no individual’s answers can be identified. The project's research records may be reviewed by departments at Purdue University responsible for regulatory and research oversight. Breach of confidentiality is always a risk with data, but we will take precautions to minimize this risk.

**What are my rights if I take part in this study?**  You do not have to participate in this research project. If you agree to participate, you may withdraw your participation at any time without penalty. If you wish to comment on any questions, please feel free to use the text box provided at the end of the survey.

**Who can I contact if I have questions about the study?** If you have any questions, comments, or concerns regarding this survey, please contact the principal investigators using this contact information: • Dr. Nicole Widmar at Purdue University by phone: (765) 494-2567 or email: nwidmar@purdue.edu (initial point of contact) • Dr. Courtney Bir at Oklahoma State University by email: courtney.bir@okstate.edu • Mario Ortez, Ph.D. Student at Purdue University by email: mortez@purdue.edu

**To report anonymously via Purdue’s Hotline see www.purdue.edu/hotline**

If you have questions about your rights while taking part in the study or have concerns about the treatment of research participants, please call the Human Research Protection Program at (765) 494-5942, email (irb@purdue.edu), or write to: Human Research Protection Program - Purdue University Ernest C. Young Hall, Room 1032 155 S. Grant St. West Lafayette, IN 47907-2114   Documentation of Informed Consent I have had the opportunity to read this consent form and have the research study explained. I have had the opportunity to ask questions about the research study, and my questions have been answered. I am prepared to participate in the research study described above. I can print a copy of this consent form for my records. By clicking the arrow below, I give my consent to participate in this study.

Q1.2 I am:

- Male (1)
- Female (2)

Q1.3 I am _____ years old.

- Under 18 (1)
- 18 - 24 (2)
- 25 - 34 (3)
- 35 - 44 (4)
- 45 - 54 (5)
- 55 - 64 (6)
- 65 + (7)

Q1.4 Please enter the number of household members - including adults and children - within each age bracket currently living in your household.    Please include yourself in the count.

|  | Total number of |
| --- | --- |
|  | household members (1) |
| Adults (over 18 years) (1) |  |
| Children ages 0 to 4 years (2) |  |
| Children ages 5 to 10 years (3) |  |
| Children ages 11 to 15 years (4) |  |
| Children ages 16 to 18 years (5) |  |

Q1.5 My annual pre-tax, household income is:

- $0-$24,999 (1)
- $25,000-$49,999 (2)
- $50,000-$74,999 (3)
- $75,000-$99,999 (4)
- $100,000 and higher (5)

Q1.6 The best description of my educational background is:

- Did not graduate from high school (1)
- Graduated from high school, Did not attend college	(2)
- Attended College, No Degree earned (3)
- Attended College, Associate's or Bachelor's Degree earned (4)
- Attended College, Graduate or Professional Degree earned (5)

Q1.8 My region of residence is: ___________. Select one option from the drop down menu.

- Northeast (CT, ME, MA, NH, NJ, NY, PA, RI, VT) (1)
- South (AL, AR, DE, DC, FL, GA, KY, LA, MD, MS, NC, OK, SC, TN, TX, VA, WV) (2)
- Midwest (IL, IN, IA, KS, MI, MN, MO, NE, ND, OH, SD, WI) (3)
- West (AK, AZ, CA, CO, HI, ID, MT, NV, NM, OR, UT, WA, WY) (4)

Q8.1 Please indicate on the following scale how impacted you were by COVID-19 in the November - December 2020 time period.

|  | 1 (Not impacted) (1) | 2 (2) | 3 (3) | 4 (4) | 5 (Impacted) (5) | Does not apply to me (6) |
| --- | --- | --- | --- | --- | --- | --- |
| Ability to buy paper products (e.g., toilet paper, paper towels) (1) |  |  |  |  |  |  |
| Ability to find meat, milk, and perishable grocery items (2) |  |  |  |  |  |  |
| Ability to execute travel plans (3) |  |  |  |  |  |  |
| Illness in my family/household from COVID-19 (4) |  |  |  |  |  |  |
| Death of family member(s) from COVID-19 (5) |  |  |  |  |  |  |
| Lack of childcare impacted my ability to work (6) |  |  |  |  |  |  |
| I took on education-related responsibilities for my child (7) |  |  |  |  |  |  |
| I took on care responsibilities for an adult member of my family (8) |  |  |  |  |  |  |

Q8.2 How likely are you to participate in the following personal behaviors in the coming 9 months?

|  | Extremely unlikely (1) (13) | 2 (14) | 3 (15) | 4 (16) | Extremely likely (5) (17) |
| --- | --- | --- | --- | --- | --- |
| Wear a mask or face covering in public (1) |  |  |  |  |  |
| Reduce number of non-essential errands/interactions around town (3) |  |  |  |  |  |
| Reduce out-of-town travel (4) |  |  |  |  |  |
| Comply with governmental orders regarding closures or lock downs (5) |  |  |  |  |  |
| Comply with government recommendations regarding social distancing (6) |  |  |  |  |  |

Q8.3 What best describes your intent or behavior towards obtaining a COVID-19 vaccine once it is made available to you?

- I have already received both doses of the vaccine (1)
- I have obtained the first shot of the vaccine and will obtain my second shot on time (2)
- I have obtained the first shot but do not plan to obtain my second shot (4)
- I have an appointment for my first shot scheduled (5)
- I am eligible to obtain the vaccine and intend to, but have not yet taken steps to do so (6)
- I am not yet eligible to obtain the vaccine, but intend to do so once I am eligible (7)
- I do not intend to obtain the vaccine (3)
- Other, please explain (8) ________________________________________________

Q9.1 Please indicate on a scale from 1 (strongly disagree) to 5 (strongly agree) your level of agreement with the following statements.

|  | 1 (strongly disagree) (1) | 2 (2) | 3 (3) | 4 (4) | 5 (strongly agree) (5) |
| --- | --- | --- | --- | --- | --- |
| Gun ownership is a right based on the U.S. Constitution (1) |  |  |  |  |  |
| Healthcare is a human right (4) |  |  |  |  |  |
| I always wear my seat belt when driving (9) |  |  |  |  |  |
| I frequently drink alcohol (10) |  |  |  |  |  |
| I frequently smoke (11) |  |  |  |  |  |
| I believe we have a societal responsibility to protect children (12) |  |  |  |  |  |
| I believe we have a societal responsibility to protect the elderly (13) |  |  |  |  |  |
| Someone in my household, or that I frequently spend time, with is at higher risk of complications of COVID-19 (14) |  |  |  |  |  |
| I am in the higher risk group for complications of COVID-19 (15) |  |  |  |  |  |

Q9.2 Please indicate if you or a member of your household experienced any of the following events since March of 2020?

|  | Yes (1) | No (2) |
| --- | --- | --- |
| Divorce (1) |  |  |
| New Marriage (2) |  |  |
| Moving/Relocation (3) |  |  |
| Death of a household or family member (4) |  |  |
| Serious illness of household or family member (5) |  |  |
| Start of a new job (6) |  |  |
| Loss of job (7) |  |  |
| Serious Financial Distress (8) |  |  |
| Pregnancy (9) |  |  |
| Purchase of a new home (10) |  |  |
